# Pararectal aggressive angiomyxoma with bilateral ovarian endometriomas cysts: a rare case report

**DOI:** 10.3389/fonc.2026.1843939

**Published:** 2026-09-17

**Authors:** Nuowei Ji, Xiangshu Kong, Jiazhen Huang, Ning Wang

**Affiliations:** Department of Gynecology and Obstetrics, Second Affiliated Hospital of Dalian Medical University, Dalian, China

**Keywords:** aggressive angiomyxoma, obstetrics and gynecology, ovarian endometriotic cyst, pelvic neoplasm, surgical resection

## Abstract

**Background:**

Aggressive Angiomyxoma (AAM) is a rare mesenchymal tumor characterized by local invasiveness and a high recurrence rate, often arising in the pelvis and perineum.

**Case presentation:**

We report a 49-year-old woman presenting with a long-standing pelvic mass. Imaging suggested bilateral ovarian endometriomas and a deep pelvic lesion suspicious for AAM. The patient underwent total hysterectomy, bilateral adnexectomy, and complete resection of a pararectal mass via a pure transabdominal laparotomy, without any perineal incision or manipulation. Histopathology and immunohistochemistry confirmed pararectal Aggressive Angiomyxoma coexisting with bilateral ovarian endometriotic cysts.

**Conclusion:**

This rare combination highlights the importance of MRI in preoperative diagnosis and the challenges of surgical management for deep pelvic AAM. Complete resection and long-term follow-up are essential to reduce recurrence.

## 1 Introduction

Aggressive Angiomyxoma (AAM) is a rare soft tissue tumor originating from mesenchymal tissue. Since its first systematic report and naming by Steeper and Rosai in 1983 (1), it has become a challenging issue in clinical diagnosis and treatment due to its extremely low incidence rate (less than 1per 1,000,000 (4)), as well as its insidious and nonspecific clinical manifestations. This tumor primarily affects women of childbearing age to perimenopausal period, with common sites of occurrence being the pelvic cavity, perineum, and vulva. Its core biological characteristic is local invasive growth—although it lacks distant metastatic potential, it is prone to infiltrating nearby important structures such as the bladder, rectum, and pelvic nerves. The postoperative recurrence rate is as high as 30% to 50% (4) (8) significantly reducing patients’ quality of life.

In clinical practice, AAM is often misdiagnosed as common pelvic lesions such as ovarian cysts, uterine fibroids, or pelvic abscesses due to the lack of specificity in symptoms. When combined with other benign pelvic diseases, the diagnosis becomes even more challenging. In this case, the patient presented with both bilateral ovarian endometriotic cysts and Pararectal Aggressive Angiomyxoma, with overlapping symptoms, leading to the AAM lesion remaining “hidden” for a long time before surgery. More importantly, when the AAM lesion is located in the deep pelvic space, intraoperative localization and complete resection are extremely challenging. Surgeons need to precisely grasp the pelvic anatomy to avoid the risk of damage to adjacent organs. In view of this, this article systematically reviews and reports in detail the entire process of diagnosis and treatment of this rare combined case, and combines relevant literature review to deeply explore the diagnostic points, surgical difficulties, and treatment strategies of AAM, with the aim of providing practical references for clinicians to enhance their understanding of the disease and optimize their diagnostic and treatment approaches.

## 2 Case presentation

### 2.1 Medical history

A 49-year-old woman was admitted to our hospital on August 18, 2025, due to a “pelvic mass detected on physical examination 4 years ago and a palpable abdominal mass for 7 months”. In 2021, a mass of approximately 7 cm in the left adnexal region was first detected on physical examination but received no treatment. Seven months prior to admission, she noticed a palpable abdominal mass. Ultrasound at an outside hospital showed bilateral adnexal masses (right: approximately 7 cm, left: approximately 8 cm), suggestive of bilateral ovarian endometriotic cysts (chocolate cysts). She was admitted to our hospital for further treatment. Her menstrual cycles were irregular (7–9/30–45 days) with moderate flow and mild dysmenorrhea (VAS score 3). She was G2P2, with two full-term vaginal deliveries in 2004 and 2015. She denied a history of chronic diseases, surgery, or infectious diseases.

### 2.2 Gynecological examination

The vulva was normal. The vagina was unobstructed with moderate, white, odorless discharge. The cervix was normal in size and smooth. The uterus was anteverted, normal in size, mobile, and non-tender. A mass of approximately 8 cm was palpable in the left adnexal region, mobile and mildly tender. A mass of approximately 6 cm was palpable in the right adnexal region, mobile and mildly tender.

### 2.3 Auxiliary examinations

#### 2.3.1 Ultrasound

The uterus was anteflexed and regular in morphology. The uterine body measured approximately 54×57×46 mm. Endometrial thickness was approximately 15 mm, and cervical length was approximately 27 mm. An anechoic lesion with dense internal echoes was observed in the right ovary, measuring approximately 68×61×55 mm with a clear boundary. A similar anechoic lesion was seen in the left ovary, measuring approximately 81×71×66 mm with a clear boundary. No obvious free fluid was detected in the rectouterine pouch.

#### 2.3.2 Pelvic MRI (non-contrast)

Bilateral adnexal cystic lesions were consistent with ovarian endometriotic cysts. A strip-shaped lesion in the right pararectal space showed slightly low signal on T1WI, slightly high signal on T2WI, and isointense signal on DWI. Pararectal Aggressive Angiomyxoma was highly suspected (**Figures 1A–G**).

**Figure 1:**
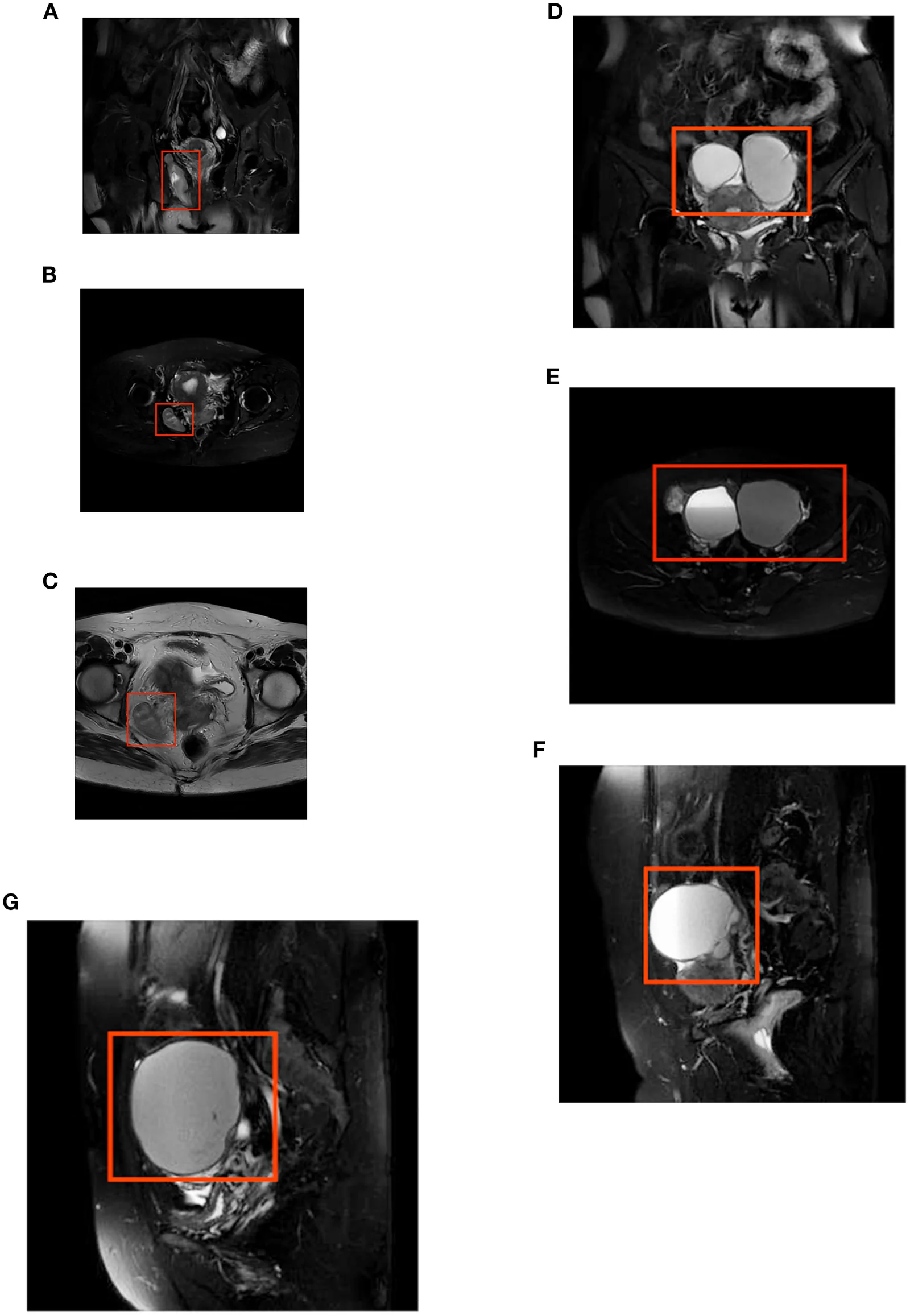
MRI. **(A)** MRI Coronal view: T1WI image, the marked area is invasive hemangiomyxoma. **(B)** MRI Axial view: T1WI image, the marked area is invasive hemangiomyxoma. **(C)** MRI Axial view: T2WI image shows invasive hemangiomyxoma. **(D)** MRI Coronal view: Bilateral ovarian endometriotic cysts. **(E)** MRI Axial view: Bilateral ovarian endometriotic cysts. **(F)** Sagittal view: An endometriotic cyst in the right ovary. **(G)** Sagittal view: An endometriotic cyst in the left ovary.

#### 2.3.3 PET-CT

Cystic mass in the right adnexal region: local thickening of the inner inferior wall with increased glucose metabolism, and mildly elevated glucose metabolism in the remaining cyst wall and cavity. Findings were most consistent with ovarian endometriotic cysts; focal malignant transformation of the cyst wall could not be excluded. Cystic mass in the left adnexal region: diffusely mildly increased glucose metabolism in the cyst wall, suggestive of a benign lesion, most likely ovarian endometriotic cysts. Soft-tissue density nodule in the right pararectal space: mildly increased glucose metabolism (decreased on delayed imaging), suggestive of a benign lesion (**Figures 2A–D**).

**Figure 2:**
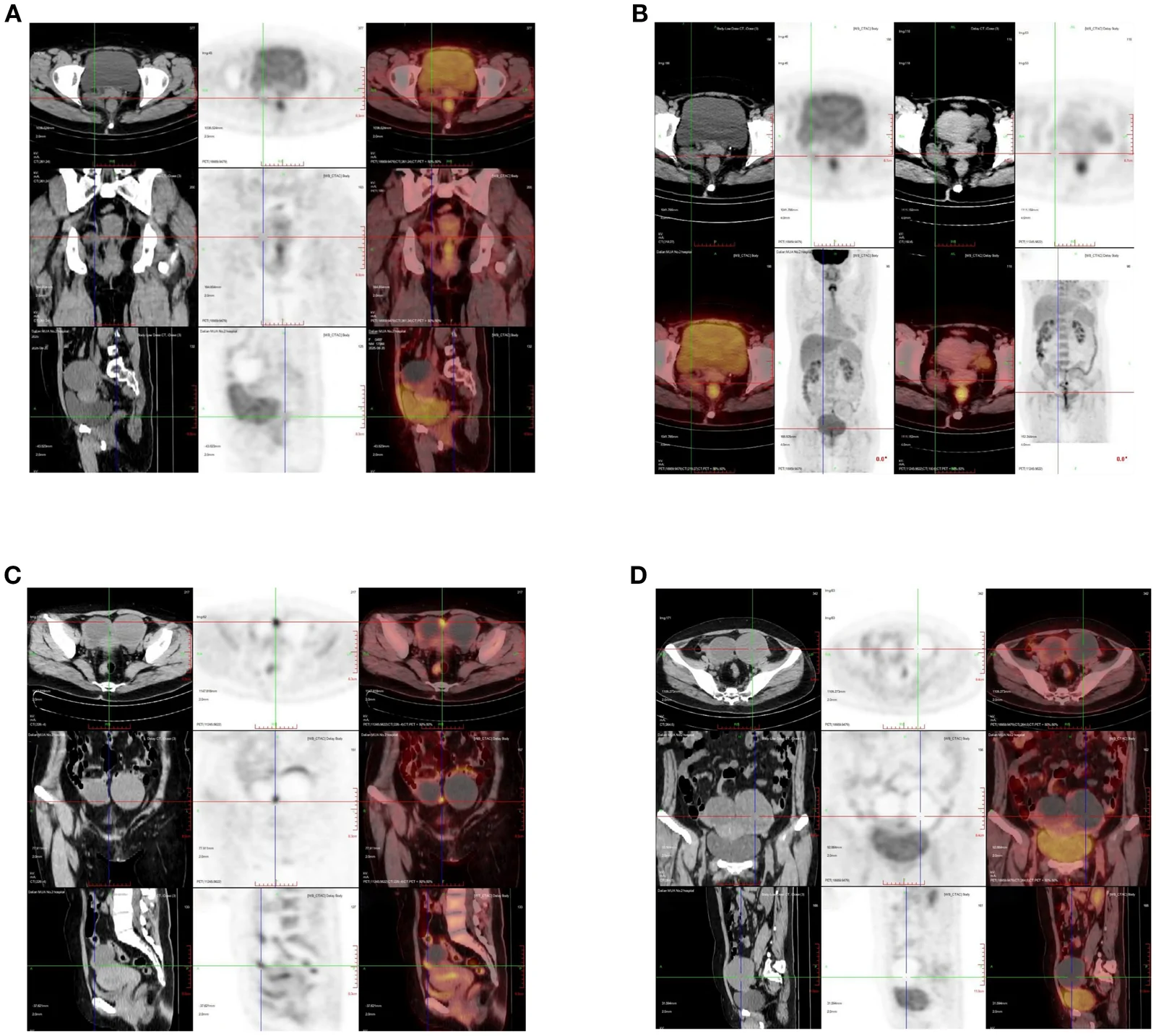
PET-CT. **(A)** Multiplanar images of PET-CT:Soft-tissue attenuation nodule in the right recto-pelvic space. **(B)** Axial view of PET-CT: Soft-tissue attenuation nodule in the right recto-pelvic space. **(C)** Multiplanar images of PET-CT: Cystic mass in the right adnexal region. **(D)** Multiplanar images of PET-CT: Cystic mass in the left adnexal region. **(A, B)** Soft-tissue density nodule between the right pelvis and the rectum, measuring approximately 41 × 23 × 23 mm, with patchy increased FDG uptake; SUVmax is approximately 2.3, and 1.3 on delayed imaging. **(C, D)** Cystic mass in the right adnexal region, measuring approximately 60 × 57 × 57 mm. Focal thickening of the cyst wall is seen at the inferior margin of the cyst, with short linear increased FDG uptake; SUVmax is approximately 4.4, and 7.0 on delayed imaging. No significant thickening is identified in the remaining portions of the cyst wall, which show mildly diffuse increased FDG uptake with a SUVmax of approximately 1.7. C. Cystic mass in the right adnexal region, measuring approximately 60 × 57 × 57 mm. Focal thickening of the cyst wall is noted at the inferior margin of the cyst, with short linear increased FDG uptake; SUVmax is approximately 4.4, and 7.0 on delayed imaging. No significant abnormal thickening is identified in the remaining portions of the cyst wall, which show mildly diffuse increased FDG uptake with a SUVmax of approximately 1.7. Focal thickening of the cyst wall at the inferior margin of the right adnexal cyst demonstrates short linear increased FDG uptake, with a SUVmax of approximately 4.4 and 7.0 on delayed imaging. **(D)** Cystic lesion in the left adnexal region, measuring approximately 74 × 76 × 84 mm. The cyst wall shows mildly diffuse increased FDG uptake with a maximum SUV (SUVmax) of approximately 1.4.

#### 2.3.4 Laboratory tests

1. Reproductive hormones: FSH 5.62 mIU/ml, LH 13.62 mIU/ml, E2 85.72 pmol/L, P 7.34 nmol/L, Testo 0.15 nmol/L, PRL 188.57 mIU/L, AMH 0.41 ng/ml.
2. Tumor markers (**Table 1**): CA19-9 1054.55 ng/ml; HE4 54.63 pmol/L; CA125 40.60 U/ml.

**Table 1:** Annotation: tumor marker measurements and reference templates.

Tumor markers	Measured value	Reference value
CA19-9 (ng/ml)	1054.55	0-30.9
CA125 (U/ml)	40.60	≤35.00
HE4 (pmol/L)	54.63	Premenopause:≤92.1; Postmenopausal:≤121

### 2.4 Treatment

#### 2.4.1 Diagnosis

(1) Pelvic mass (Pararectal Aggressive Angiomyxoma)?; (2) Bilateral ovarian tumors (ovarian endometriotic cysts? Ovarian carcinoma)?; (3) Intrauterine device (removed).

#### 2.4.2 Surgery and pathology

After detailed multidisciplinary discussion and full informed consent with the patient and her family regarding the benefits and risks of treatment options, a treatment plan was formulated. The patient’s CA19–9 was significantly elevated, and PET-CT could not exclude malignant transformation of the bilateral ovarian masses. Given that the patient was 49 years old and had completed childbearing, exploratory laparotomy via a pure transabdominal approach (without any perineal incision or manipulation) was recommended, including total hysterectomy and bilateral adnexectomy. Intraoperative inspection of the cysts and frozen section examination would be performed if necessary; if malignancy was confirmed, the surgical scope would be extended.

Intraoperative exploration revealed a cystically enlarged right ovary (7×6×6 cm) with a grayish-white surface, black and dark red nodules inside, and dense adhesions to the uterine body and anterior rectal wall. The left ovary was cystically enlarged (8×7×7 cm) with a grayish-white surface and dense adhesions to the posterior leaf of the left broad ligament, left posterior uterine wall, and rectum (**Figure 3A**). Both fallopian tubes were normal in morphology. Due to dense adhesions, sharp and blunt dissection were combined to carefully release adhesions and restore pelvic anatomy while avoiding injury to the rectum and uterine vessels. Both cysts contained chocolate-like fluid consistent with bilateral ovarian endometriotic cysts. Intraoperative frozen sections were obtained from both lesions and reported as benign (**Figures 4B, C**). After total hysterectomy and bilateral adnexectomy, the pararectal lesion was identified according to MRI findings and confirmed by intraoperative digital rectal examination. The mass was located in the right inferior rectum near the pelvic wall, a deep site surrounded by the pelvic plexus and piriformis, resulting in poor surgical exposure. For precise localization, the right lateral peritoneum was opened with electrosurgery and dissection was carried out inferiorly along the right pelvic wall. The pelvic plexus and piriformis were carefully identified and preserved. After removing surrounding fat and connective tissue, a mass of approximately 5×3 cm was palpated in the deep space, which was grayish-white, soft, and had an intact capsule (**Figure 3B**). The mass and involved pelvic peritoneum was completely dissected and resected while maintaining stable pelvic anatomy (**Figure 4A**). Post-resection digital rectal examination confirmed no residual tumor or rectal injury. The operation was completed smoothly with an estimated blood loss of approximately 200 ml.

**Figure 3:**
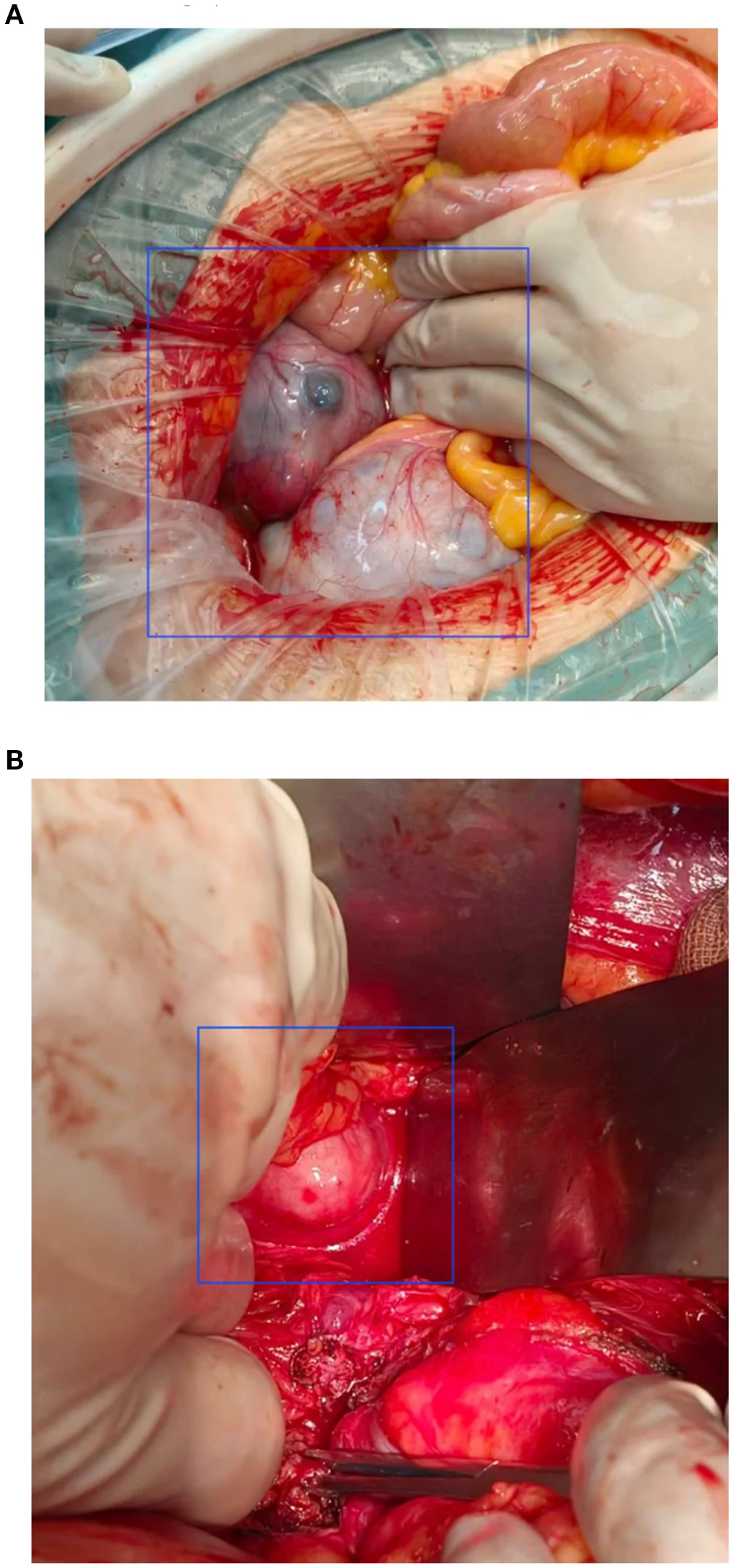
Intraoperative findings **(A)** Bilateral ovarian endometriotic cysts. **(B)** Aggressive angiomyxoma.

**Figure 4:**
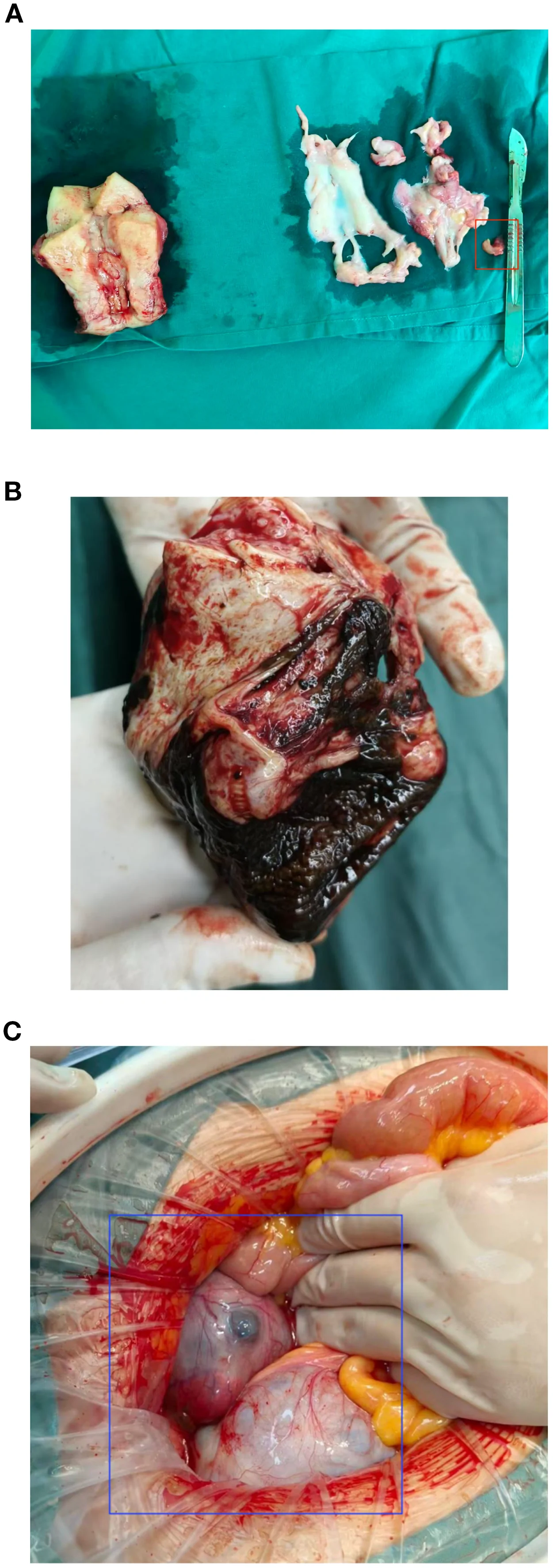
Resected specimen. **(A)** Intraoperatively resected aggressive angiomyxoma. **(B)** Intraoperatively resected left adnexa. **(C)** Intraoperatively resected right adnexa.

Postoperative paraffin pathology: (Right adnexa) right ovarian endometriotic cyst; (Left adnexa) left ovarian endometriotic cyst; (Pelvic floor mass) spindle cell tumor, consistent with pararectal Aggressive Angiomyxoma based on immunohistochemistry. One sampled lymph node showed no evidence of malignancy. Immunohistochemistry: spindle cells CD10 (focal +), Calponin (+), ER (+), PR (+), Desmin (+), SMA (−), S-100 (−), CD34 (−), β-catenin (focal cytoplasmic +), Ki-67 (+, approximately 1%), ALK80 (−).

### 2.5 Outcome and follow-up

The patient recovered well postoperatively with a good general condition and was discharged smoothly on postoperative day 3. No obvious abnormalities were detected on pelvic MRI and triple examination at 4 months after surgery. Regular follow-up was recommended.

## 3 Discussion

### 3.1 Epidemiology, clinical features and prognosis

Aggressive Angiomyxoma was first reported by Steeper and Rosai in 1983 as a rare tumor derived from mesenchymal tissue (1). It predominantly affects women of reproductive and perimenopausal age, with typical locations in the pelvic cavity, perineum, and vulva, and an incidence of less than 1 per 1,000,000 (2). The tumor exhibits unique biological behavior: although it lacks distant metastatic potential, it is locally invasive and often invades adjacent structures such as the bladder, rectum, and pelvic nerves, with a postoperative recurrence rate as high as 30%–50% (3, 4). Most patients present with a painless pelvic mass. Some patients may develop dysuria due to urethral compression or dyspareunia due to vaginal wall invasion as the tumor enlarges. In recent years, Espejo-Reina et al. have reported cases of women in the third trimester presenting with genital tract prolapse as the initial manifestation, suggesting that clinical manifestations are highly diverse and easily confused with common benign gynecological lesions such as Bartholin gland cysts, pelvic abscesses, or submucous uterine fibroids (5, 6). This is highly consistent with the conclusion proposed by Figueiredo et al. in their study of rare pelvic mesenchymal tumors that non-specific clinical manifestations are the main cause of misdiagnosis (7). In this case, the pararectal Aggressive Angiomyxoma lesion was located in the deep pelvic pararectal space, which is more insidious and less frequently reported than the vulva and vagina (8, 9). This case is clinically unique due to the coexistence of pararectal Aggressive Angiomyxoma and bilateral ovarian endometriotic cysts. The consistent positive expression of ER and PR in pararectal Aggressive Angiomyxoma reflects the hormone dependence of AAM, and endometriosis is also a hormone-dependent disease. Although no other literature has reported the coexistence of these two diseases, whether their pathogenesis is related requires further exploration. This is highly consistent with the diagnostic and therapeutic difficulties of this disease summarized by Bai HM et al. (10).

### 3.2 Diagnosis

For diagnosis, imaging examination is critical for accurate preoperative evaluation. Studies by Pan Zhenyu et al. further confirmed the superior value of MRI in the qualitative diagnosis of lesions. Typical MRI features include homogeneous high signal on T2WI (related to myxoid matrix), isointense or slightly low signal on T1WI, and no obvious restricted diffusion on DWI (9). In this case, initial symptoms and ultrasound findings were consistent with bilateral ovarian endometriotic cysts. The pararectal Aggressive Angiomyxoma was not detected by ultrasound due to its deep location and relatively small size. PET-CT only indicated that the pararectal nodule was a benign lesion and could not determine the specific pathological type. Only MRI raised the possibility of AAM based on the typical features of slightly low signal on T1WI, slightly high signal on T2WI, and isointense signal on DWI (4) (11). The muscular and tissue structures of the pelvic floor are complex. MRI offers advantages in the diagnosis of AAM due to its multi-sequence, multi-planar, and high-resolution characteristics, allowing multi-angle imaging including sagittal and coronal planes to more clearly identify the origin of AAM. This imaging evaluation overcomes the limitations of ultrasound, not only suggesting the possibility of the disease but also providing crucial intraoperative guidance for subsequent surgery, greatly assisting surgeons in determining the exact location and extent of AAM. This is consistent with published reports: MRI, with high soft-tissue resolution, can clearly demonstrate the boundaries, internal structure, and relationship of AAM with surrounding tissues, making it the most valuable imaging modality for preoperative diagnosis of AAM.

Some studies suggest that immunohistochemical features reveal a relatively specific marker expression profile for AAM. ER and PR are expressed in 83.3%–100% and 80%–91.7% of cases, respectively. In addition to ER and PR, vimentin, desmin, and SMA are usually positive, while S-100 is negative (9). However, a study by Xue-Mei Lin et al. searched the PubMed database and retrieved more than 500 cases and approximately 300 eligible articles from 1983 to 2020. The authors noted that the pathological morphology of AAM may resemble other mesenchymal lesions, posing diagnostic challenges for pathologists. AAM masses described in the literature are usually deep, ill-defined, and range from 1 to 60 cm in diameter. Microscopically, the tumor consists of a myxoid matrix with numerous blood vessels of varying calibers and types scattered throughout. Tumor cells are sparsely and uniformly distributed, spindle-shaped and/or stellate, without mitotic activity or nuclear atypia. Delicate smooth muscle bundles are often observed around blood vessels, which is considered a characteristic feature of AAM (12). Tumor cells show variable expression of vimentin, connexin, CD34, SMA, ER, and PR, but these markers are not specific for the pathological diagnosis of AAM. The study by Xue-Mei Lin et al. also noted that when the pathological morphology of AAM overlaps with other mesenchymal lesions, a comprehensive evaluation of clinical, pathological, and imaging features is essential for accurate diagnosis of AAM. Differential diagnoses include angiomyofibroblastoma, fibroepithelial stromal polyp, superficial angiomyxoma, cellular angiofibroma, and superficial cervicovaginal myofibroblastoma, based on location, boundary, tumor size, morphology, cell density, matrix, mitotic activity, blood vessels, and immunophenotype (12). Therefore, in clinical practice, pelvic MRI should be performed if a pelvic mass is detected on bimanual or triple examination but is not clearly diagnosed by ultrasound. In this case, CA19–9 was significantly elevated, and no obvious abnormalities were found in the biliary tract, pancreas, stomach, or intestines on PET-CT. Studies have shown that tumor marker levels vary by subtype: CA19–9 is higher in ovarian endometriotic cysts, and CEA is higher in deep endometriosis (13). The elevated CA19–9 in this case was associated with ovarian endometriotic cysts.

### 3.3 Treatment and surgical challenges

At present, extensive surgical resection remains the first-line treatment for AAM (6). Achieving complete tumor resection with negative surgical margins is the key to reducing the recurrence rate. However, since AAM is often located in the pelvic cavity and perineum with indistinct borders and invasive growth, it frequently involves multiple organs and vital structures, making complete resection difficult (12, 14). For patients with unresectable lesions, maximal cytoreductive surgery should be performed to relieve symptoms and create conditions for subsequent treatment. In this case, intraoperative localization of the pararectal Aggressive Angiomyxoma mass was extremely challenging. The lesion was anatomically located in the right inferior rectum near the pelvic wall, within a narrow space formed by the pelvic plexus, piriformis, and iliac vessels. The surrounding anatomy was complex and surgical exposure was poor. The patient had extensive pelvic adhesions secondary to bilateral ovarian endometriotic cysts, resulting in unclear boundaries between the uterus, ovary, and rectum, which further increased the difficulty of lesion localization. As a soft-tissue tumor, AAM has no obvious bony landmarks and can only be initially localized by preoperative imaging and intraoperative digital rectal examination.

Preoperative MRI localization combined with intraoperative digital rectal examination facilitates accurate localization of deep-seated tumors. Therefore, we adopted a stepwise surgical strategy. Based on MRI findings, we clarified the relative position of the lesion to the rectum and pelvic plexus and formulated a surgical plan via the lateral peritoneal approach. We first resected the bilateral adnexal cysts and uterus, released pelvic adhesions, and restored partial anatomy to create space for subsequent lesion exploration. Combined with MRI images, intraoperative digital rectal examination (to define the general extent of the lesion), and direct visualization dissection (dissecting gradually along the course of the pelvic plexus), we accurately identified the lesion. Strict aseptic technique was applied: sterile gloves and surgical gowns were replaced immediately after touching the rectum to avoid surgical field contamination. These strategies ultimately achieved complete resection of the lesion without injury to adjacent nerves, blood vessels, or the rectum. This is consistent with the concept of “multimodal therapy to avoid destructive surgery” proposed by Han-Geurts et al., providing a reference for surgical management of similar cases (6).

In addition to surgical resection, the value of hormonal therapy for this tumor has been supported by multiple high-level studies. An international multicenter study led by Fucà included 126 patients and confirmed that tumor cells are highly sensitive to hormonal agents such as tamoxifen and gonadotropin-releasing hormone agonists (GnRH-a) due to high expression of ER and PR, with an efficacy rate of over 60% (15). Hormonal therapy is particularly suitable for patients with postoperative recurrence, unresectable tumors, or young patients with fertility needs (4). Pannier et al. also confirmed the potential of hormonal therapy for this tumor (15). A recent study by Maciejczyk in 2025 further demonstrated that sequential therapy with GnRH-a combined with aromatase inhibitors can prolong disease control. Besides surgery and hormonal therapy, radiotherapy has also been attempted as an adjuvant treatment in some cases, especially for unresectable or recurrent cases. However, large-sample studies are lacking to confirm its long-term efficacy. For refractory cases, targeted therapy may represent a future research direction, but relevant studies are limited and not yet widely used in clinical practice (14). Although GnRH-a was not used in this patient, bilateral oophorectomy reduced postoperative estrogen and progesterone levels, thereby lowering the risk of postoperative recurrence. This suggests that perimenopausal women may benefit from bilateral oophorectomy at the time of Aggressive Angiomyxoma resection.

### 3.4 Prognosis and follow-up

For prognosis evaluation, long-term follow-up data of 27 patients over 14 years from a single center reported by Zou et al. showed that the 5-year postoperative recurrence rate of this tumor was as high as 30%–50%, and 80% of recurrences occurred within 2 years after surgery. Most recurrences were located around the primary lesion or in the deep pelvic fascial space (9). Therefore, long-term regular postoperative follow-up is crucial. MRI is recommended as the main follow-up modality, with contrast-enhanced pelvic MRI every 6–12 months, combined with gynecological examination and tumor marker monitoring. The follow-up period should last at least 5 years. This forms a complete diagnosis and treatment system with the preoperative evaluation and postoperative follow-up recommendations proposed by Pan Zhenyu et al (9, 11).

### 3.5 Summary and clinical significance

In summary, this case of pararectal Aggressive Angiomyxoma combined with bilateral ovarian endometriotic cysts is extremely rare. Preoperative diagnosis relies on accurate imaging examinations such as MRI. Intraoperative localization and resection of the lesion are extremely challenging due to its deep anatomical location and coexisting adhesions. This case not only further enriches the clinical case database of Aggressive Angiomyxoma but also enhances the understanding of the tumor’s insidious and diverse clinical manifestations, typical imaging features, and specific pathological immunohistochemistry. Accurate evaluation of tumor extent via multimodal imaging before surgery, complete resection of the lesion, and close long-term follow-up are the keys to establishing a comprehensive diagnosis and treatment system for this disease and improving patient prognosis (4, 7, 11). In the future, more prospective cohort studies are needed to explore the molecular mechanisms underlying its pathogenesis and more optimized targeted therapeutic regimens. With further research, new breakthroughs in the diagnosis and treatment of AAM are expected.

## Data Availability

The original contributions presented in the study are included in the article/supplementary material. Further inquiries can be directed to the corresponding authors.
